# IOTEML: An Internet of Things (IoT)-Based Enhanced Machine Learning Model for Tumour Investigation

**DOI:** 10.1155/2022/1391340

**Published:** 2022-09-14

**Authors:** B. Swaminathan, Siddhartha Choubey, N. K Anushkannan, Jeevanantham Arumugam, K Suriyakrishnaan, Hesham S. Almoallim, Sulaiman Ali Alharbi, S. R. Soma, Ramata Mosissa

**Affiliations:** ^1^Department of Computer Science and Engineering, Saveetha School of Engineering, Chennai, Tamil Nadu 602105, India; ^2^Department of Computer Science and Engineering, Shri Shankaracharya Technical Campus, Durg, Chhattisgarh 491001, India; ^3^Department of Electronics and Communication Engineering, Kathir College of Engineering, Coimbatore, Tamil Nadu 641062, India; ^4^Department of Information Technology, Kongu Engineering College, Perundurai, Tamil Nadu 638060, India; ^5^Department of Electronics and Communication Engineering, Sona College of Technology, Salem, Tamil Nadu 636005, India; ^6^Department of Oral and Maxillofacial Surgery, College of Dentistry, King Saud University, PO Box 60169, Riyadh 11545, Saudi Arabia; ^7^Department of Botany and Microbiology, College of Science, King Saud University, PO Box 2455, Riyadh 11451, Saudi Arabia; ^8^Department of Sciences, University of Tennessee Health Science Center, Memphis, USA; ^9^Department of IT, Mettu University, Metu, Ethiopia

## Abstract

In the current age of technology, various diseases in the body are also on the rise. Tumours that cause more discomfort in the body are set to increase the discomfort of most patients. Patients experience different effects depending on the tumour size and type. Future developments in the medical field are moving towards the development of tools based on IoT devices. These advances will in the future follow special features designed based on multiple machine learning developed by artificial intelligence. In that order, an improved algorithm named Internet of Things-based enhanced machine learning is proposed in this paper. What makes it special is that it involves separate functions to diagnose each type of tumour. It analyzes and calculates things like the size, shape, and location of the tumour. Cure from cancer is determined by the stage at which we find cancer. Early detection of cancer has the potential to cure quickly. At a saturation point, the proposed Internet of Things-based enhanced machine learning model achieved 94.56% of accuracy, 94.12% of precision, 94.98% of recall, 95.12% of *F*1-score, and 1856 ms of execution time. The simulation is conducted to test the efficacy of the model, and the results of the simulation show that the proposed Internet of Things-based enhanced machine learning obtains a higher rate of intelligence than other methods.

## 1. Introduction

The lung neuroendocrine tumour is a rare neuroendocrine tumour that develops in the lungs. It makes up 20% of all neuroendocrine tumours. This cancer is usually diagnosed in people between the ages of 45 and 60; however, it can occur at any age [1]. It is more common in white people compared to other ethnic groups. The 5-year survival rate for lung neuroendocrine tumours that have spread to other parts of the body is 58%. Lung neuroendocrine tumour involves the proliferation of certain genetic mutations. Some genes, such as TP53, MLLT3, IL21R, UIMC1, and ARID1A, are known to have genetic abnormalities in this cancer, resulting in altered function [2]. Genetic abnormalities found in lung neuroendocrine tumours can affect specific biochemical pathways that cause these cancer cells to move to invasive growth and escape from ongoing treatment. In addition, lifestyle conditions or habits such as smoking, alcohol consumption, and body mass index (BMI) can have a major impact on your doctor's response to prescribed treatments for pulmonary neuroendocrine tumours [3]. Nutrition/diet: numerous studies continue to show that both diet and nutritional supplements can adversely affect or support treatment or play no role. Therefore, the triangle of lung neuroendocrine tumour genetics, planned or ongoing treatments, and lifestyle should be thoroughly considered when designing a diet/nutrition plan to decide what foods and supplements to consider and avoid. In the current complex medical environment, more serious medical diseases such as brain tumours and some major tumours, such as lung tumours, are considered to be at high risk [4]. Also, it should be noted that when any of these conditions change, such as treatment or lifestyle or cancer diagnosis, it can affect nutrition, and the cancer nutrition program may need to be redesigned. This figure would be confusing if you take into account the countless number of people who are infected with the virus but do not show symptoms such as weakness. A test to develop immunity can help researchers determine whether a person is susceptible to disease. The British government's scientific advisers said it was unclear whether climate change would have an impact. If there is such an effect, they assume it will be less than the effect of colds and flu. If the number of coronavirus cases decreases significantly in the summer, there is a risk that its number will increase rapidly again in the winter season. It is said to be a time when hospitals are dealing with more patients due to typical winter illnesses.

Tumour sequencing provides insight into changes in patient tumour genes. Tumour DNA sequencing can also be referred to as genetic specification or genetic testing. Sequence results will enable clinical decision-making to develop a personalized cancer treatment plan based on the molecular characteristics of the tumour rather than a one-size-fits-all treatment approach. Tumour sequencing also plays a major role in cancer research [5]. Thanks to advances in human genome sequencing and tumour sorting technology since 2003, we have large databases of cancer/tumour gene sequences of populations with different types of cancer for analysis in the public domain. Analysis of these datasets of cancer (tumour) gene sequences has revealed that the genetic makeup of each patient is different and that no two cancers are identical. However, the analysis also shows that a specific tissue primary cancer, such as lung cancer or colon cancer or myeloma, may have some dominant characteristics that are unique to that type of cancer. Cancer can be defined based on the quality and stages of abnormal growth of cells. Although cancer seems dangerous the first time we hear it, how dangerous it is determined by understanding its different stages of tumour growth. Information on the size of the tumour and whether it has spread to other areas from where it originated helps to determine this.

Racial differences are found in cancers of the same origin, e.g., differences are found in the subtypes of lung cancer between the Jewish and Chinese populations. Because of these large variations in cancer characteristics, a one-size-fits-all treatment may not be a good choice for cancer patients [6]. Once a patient is diagnosed with cancer, the stage of the cancer is determined based on the size and spread of the tumour. Regular treatment options are discussed and recommended according to the guidelines. The first line option is to use specific chemotherapy for specific types of cancer. Chemotherapy can be used before tumour shrinkage surgery, it can be used to control the rapid growth of the tumour, it can be used when the tumour has spread to other parts of the body, or it can be used after surgery to clear the remnants of cancer. However, as evidenced by clinical studies, the response rate of most chemotherapy does not exceed 50–60%, due to variation in tumour genes in cancer patients [7]. Diagnosing the stages of cancer is one of the most important things we know about the severity and progression of cancer. Cancer is classified into four categories from stage 1 to stage 4. Each stage is based on detailed information about the tumour and where in the body cancer has developed. Cancer becomes more serious with each stage.

Although chemotherapy is an important area of cancer treatment and there is a need to control the rapidly growing cancer despite the severe and debilitating side effects, the choice of chemotherapy should be personalized. It provides sequential insights into changes in a patient tumour gene. The tumour sorting results help clinicians make clinical decision-making and personalized cancer treatment plans. Tumour continuity plays an important role in the development of novel targeted treatments for cancer [8]. Personalized cancer treatment is a one-size-fits-all approach to treatment that is determined by individual tumour characteristics, identified by tumour alignment, to improve the quality of life, without causing parallel damage to targeted normal cells. In addition, chemotherapy can further enhance the success and well-being of a cancer patient when it is scientifically supplemented (identified by tumour sequence) with the right natural supplements selected based on chemo and cancer characteristics [9]. Knowing what stage the cancer is in can be very helpful in treating it. This helps the doctor to determine the treatment needed to cure cancer. This will help determine whether surgery is needed or whether chemotherapy is sufficient.

Cancer genetic/DNA sequencing can help to make more accurate cancer diagnoses, better prognosis, and identify customized treatment options based on cancer's genetic characteristics. However, despite the growing popularity and exaggeration of the benefits and uses of the cancer gene sequence, there are currently only a handful of patients who benefit from it. Tumour genetic sequencing is the technique of obtaining a molecular scan of the type of DNA extracted from tumour cells obtained from a biopsy specimen or the patient blood or bone marrow. Diagnosing the stages of cancer helps researchers find more effective treatments. The research will be done in comparison with the data of the general previous patients of this study. This information provides information on which parts of tumour DNA differ from those of nontumour DNA, and the interpretation of genetic sequencing data provides insight into the key genes and drivers of cancer. More recently, the severity of the disease and the complications it causes have made the disease even more problematic. This type of tumour is the 10th most deadly disease in the world [10–15]. There have been significant advances in sequencing technologies that make tumour genetic information cheaper and more accessible for clinical use. Numerous research projects funded by various governments around the world compile data on the tumour genetic sequences of numerous cancer patients, along with their clinical history, treatment details, and clinical outcomes that are available for analysis in the public domain [16–19].

In this paper, an improved algorithm is developed named Internet of Things (IoT) based on enhanced machine learning. The major contribution of the proposed model is to analyze and calculates things like the size, shape, and location of the tumour. The current analysis of these large cancer population datasets has provided key insights into the changing landscape of cancer treatment protocols worldwide. Cancers of specific tissue origin, such as all breast cancers or all lung cancers, which were previously considered historically similar and treated the same way, are now recognized as very different and are classified as distinct molecular subtypes that need to be treated differently.

## 2. Related Works

The authors of [1] developed a hybrid fuzzy brain-storm optimization algorithm. This algorithm designed and classified the MRI scan images based on the brain tumour. Its improved various methods accurately calculated the location and shape of tumours based on brain function and its measurements. This work has [3] calculated the number of patients with tumours in the United States-based population. This means that a total of more than 7 lakh people are reported to be living everyday life with different types of tumours, and they estimate that 80% of them are benign tumours and the other 20% are malignant tumours. This work [4] released some data based on current opinion polls. According to the latest estimates, 80,000 people are affected by brain tumours. 55,000 of them are classified as belonging to types 1 and 2. Further 25,000 people are reported to be affected by type 3 and type 4 tumours. The paper [5] further simplified the computation of tumours. Evolving technologies are increasingly making it easier to calculate and classify tumours. And, the rise of IoT-based achievements has created a major industrial revolution in this modern age and has made the series of health structures even more special.

In [6], computed tomography was performed on different types of brain tumours. They examined and evaluated their series of health conditions. The status of the tumour and the risks associated with its condition can be calculated by these procedures. The authors of [7] designed a model based on simple processes that classify brain tumours. The nature and severity of the disease were diagnosed and analyzed based on the data in its proposed manner. Its improved procedure and computations accurately calculated the classification of tumours.

The authors of[8] proposed some improved methods for differentiating and analyzing the types of tumours in the gallbladder. It was designed based on the process of calculating the structure of tumours from MRI images using specific individualization processes. To confirm the tumour accuracy of this design, they approved more than 300 MRI scans of 14 patients with different tumours. This process showed the images alone and the functions of the brain alone. The authors of [9] designed an improved algorithm based on K-means. With the help of histogram technology, different categories of tumours were classified, and tumour locations were calculated based on their normal functioning. The authors of [10] developed a spontaneous artificial intelligence system. Its artificial intelligence technology was compared with various previous data to determine the status of the tumour and its type. This work [11, 12] introduced the *C*-means method based on a modified mean-change to calculate the mean size and type of tumours. It was used to obtain blurry images as input and remove unwanted parts to accurately locate tumours, and its reliability was high because its computational speed was consistent and fast.

## 3. Proposed System

**Figure 1:**
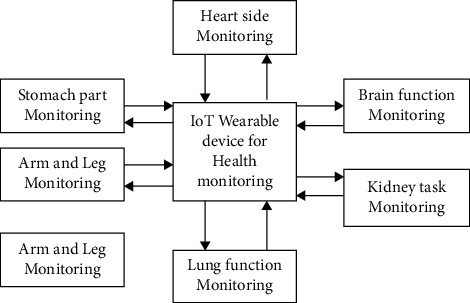
IoT-based body tumour monitoring.

The proposed IoT-based enhanced machine learning algorithm was structured, even within the molecular subtype of a particular cancer symptom, and the tumour genetic profile of each individual is different and unique. The genetic analysis of cancer DNA provides information on the major genetic abnormalities (mutations) that drive the disease, and many of these contain specific drugs designed to inhibit their action. The abnormalities of cancer DNA help to better understand the basic mechanisms by which the cancer cell uses its continuous and rapid growth and proliferation and to discover new and more targeted drugs. Therefore, in the case of a disease such as cancer, which is associated with morbidity and mortality, every piece of information that helps to understand the cancer characteristics of the individual will be useful. Listed as follows are the first three reasons why patients should order their DNA and consult a specialist with their results as in Figure 1:

  Grade 1: cancer cells look like normal cells and do not grow.
  Grade 2: cancer cells do not look like normal cells. At this level, cancer cells grow rapidly.
  Grade 3: cancer cells look abnormal. And, those cells are likely to grow or spread aggressively to other organs.

### 3.1. HLPS with a Cancer Genetic Sequence Cautious Diagnosis

**Figure 2:**
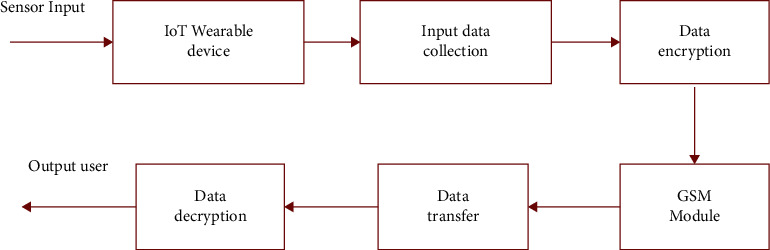
IoT wearable device data transfer to the end-user.

In many cases, the site and cause of the primary cancer are not clear, and the genetic sequence of the DNA helps to better identify the primary tumour site and the major cancer genes, thereby providing a more accurate diagnosis. For rare cancers or cancers that are late diagnosed and spread by various organs, an understanding of the cancer characteristics can help determine the most appropriate treatment options as in Figure 2.

### 3.2. HLPs with a Genetic Sequence of Cancer and Excellent Prognosis

**Figure 3:**
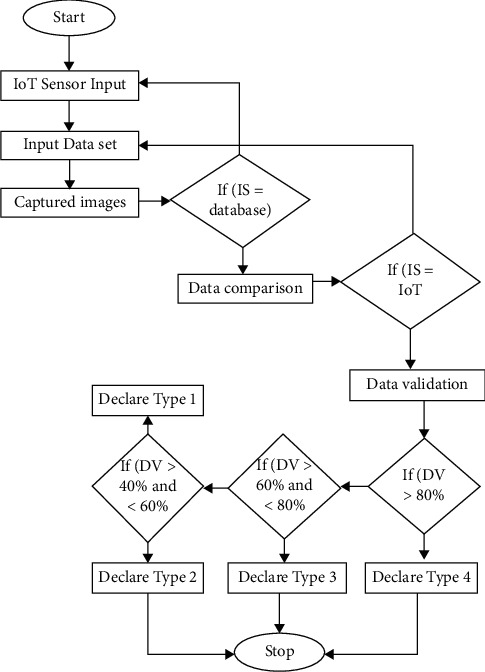
Proposed flow chart.

From the sorted data, one obtains the genetic profile of the cancer DNA. Based on the analysis of cancer demographic data, patterns of various abnormalities are associated with disease severity and treatment response. For instance, the absence of the MGMT gene predicts a better response to DMZ in patients with brain cancer multiform. The presence of a TET2 gene mutation increases the likelihood of patients with leukemia responding to a specific type of drug called agents. So, this information provides insight into the severity and characteristics of the disease and helps to select mild or more aggressive treatment. In general, some specific symptoms can be computed to diagnose tumours. Based on that data, its status and classifications are determined. Its common symptoms are classified in Table 1.  Figure 3 shows the proposed flow chart.

#### 3.2.1. IoT Indication (IoTI)

For every IoT sensor indication, a distinct rate is located to evaluate through the experiential rate as follows:

(1) $$\begin{array}{c} \text{IoT indication}\left(\text{IoTI}\right)=\left\{\begin{array}{cc} 1, & \text{indicated value }\geq \text{highly declared value},\\ 1, & \text{indicated value }<\text{low declared value},\\ -1, & \text{all other indiations}, \end{array}\right. \end{array}$$

(2) $$\begin{array}{c} \text{lack of synchronization between arm and leg}=\frac{\text{no}.\text{ of total walking foot work per day}}{\text{time taken to complet the activity}}. \end{array}$$

Cancer is classified into five stages depending on the tumours. However, other types of cancer, such as leukemia, lymphoma, and brain cancer, have different staging systems. But all stages help determine the depth of cancer:

  Stage 0: in stage 0, it is said that no cancer cells have formed till now. However, abnormal cells in body organs may have the potential to become cancer cells. This is called carcinoma.
  Stage I: this indicates small-scale cancer. It is formed in only one part of the body. This stage is often called the early stage of cancer.
  Stage II: stage II indicates that cancer has grown larger than stage I. But it did not spread to other areas.
  Stage III: if a person has stage III cancer, it means that cancer has spread to a large extent. It also indicates that cancer has spread to nearby areas.
  Stage IV: stage IV cancer is called advanced or metastatic cancer. In this case, the cancerous tumour has spread to other parts of the body. This is the final phase.

### 3.3. Genetic Sequencing of Cancer HLPs to Identify an Individual Treatment Option

For many cancer patients who do not respond to the standard of maintenance chemotherapy treatment, tumour sorting can help better identify major abnormalities that can then be treated with more recently developed target drugs and used only in specific cases. It has the required attribute. In stubborn, recurrent, and resistant cancers, the genetic specification of the tumour DNA will facilitate access and inclusion in clinical trials testing new and innovative target drugs or finding unique alternative and customized drug options (treatments) based on cancer characteristics. The grade of cancer is determined by the appearance of the cancer cells seen under a microscope. A low grade indicates slow-growing cancer, while a high grade indicates fast-growing cancer cells. The indication measurements based on Table 1 are demonstrated.

(3) $$\begin{array}{c} \text{Measurement of indication}=\left\{\begin{array}{cc} 1, & \sum_{1}^{a}\text{IoT indication value }\geq 0,\\ -1 & \text{others,} \end{array}\right. \end{array}$$

where *a* = probability constant for all related IoT indication values to the general indication.

After measuring the indication values, the detection of tumour is very simple.

(4) $$\begin{array}{c} \text{Chances for tumour}=\frac{1}{1+\;e^{-b}}. \end{array}$$

where *b* = sum of all the general indication values.

The chances of tumour probabilities obtained from the estimation are presented in Table 2, and Algorithm 1 shows the IoT-based machine learning.

## 4. Results and Discussion

The proposed IoT-based enhanced machine learning was compared with the existing hybrid fuzzy brain-storm optimization algorithm (HFBSO), multifractal texture estimation (MTE), and tumour segmentation using chi-square fuzzy c-mean clustering (CSFCC), and hybrid feature extraction method (HFEM). 5 parameters are evaluating the water quality, that is, tumour accuracy, tumour precision, tumour recall, tumour F1-score, and computation time. Before understanding the quality rate of the parameters, we will know about the following:

  Positive-*T* (TP): it is the perfect predicted correct or above the calibration level
  Negative-*T* (TN): it is the negative prediction value below the calibration level
  Positives-*F* (FP): when the exact values are at calibration level and the predicted samples are at the same level
  Negative-*F* (FN): when the exact values are at calibration level but the predicted samples are at a different level

### 4.1. Computation of Tumour Accuracy

Tumour accuracy is the parameter which describes the ratio between perfectly predicted tumour input images from the given samples to the total number of collected image samples as in equation (5). When the rate of tumour accuracy is high, then the given output image sample gets a high-quality rate.

(5) $$\begin{array}{c} \text{Tumour accuracy measurement}=\frac{\text{TP}+\text{TN}}{\text{all collected samples}}. \end{array}$$

**Figure 4:**
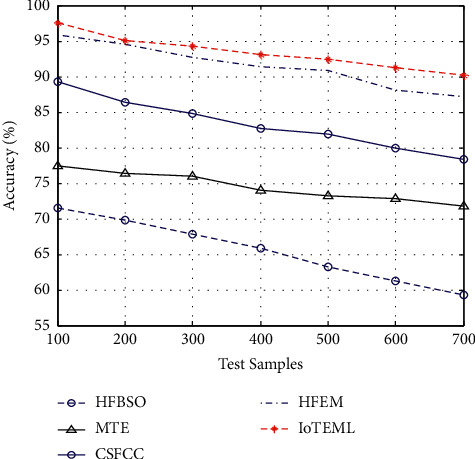
Measurement of tumour accuracy.

Figure 4 demonstrates the various measurement comparison of the tumour accuracy values between the existing HFBSO, MTE, CSFCC, HFEM, and proposed IOTEML.

### 4.2. Computation of Tumour Precision

Tumour precision measurement is the ratio between the positive true samples and total true samples as in equation (6). The total true samples are calculated by the sum of positive true samples and false positive samples.

(6) $$\begin{array}{c} \text{Tumour precision measurement }=\;\frac{\text{true positive predictions}}{\text{true positive prediction}+\text{false positive prediction}}. \end{array}$$

**Figure 5:**
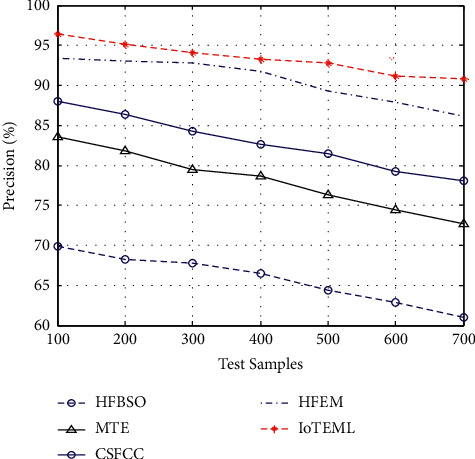
Measurement of tumour precision.

Figure 5 demonstrates the various measurement comparison of the tumour precision values between the existing HFBSO, MTE, CSFCC, HFEM, and proposed IOTEML.

### 4.3. Computation of Tumour Recall

Tumour recall measurement is the ratio between the positive true samples and the sum of positive true samples and false negative true samples:

(7) $$\begin{array}{c} \text{tumour recall measurement}=\frac{\text{true positive predictions}}{\text{true positive predictions}+\text{false negative predictions}}. \end{array}$$

**Figure 6:**
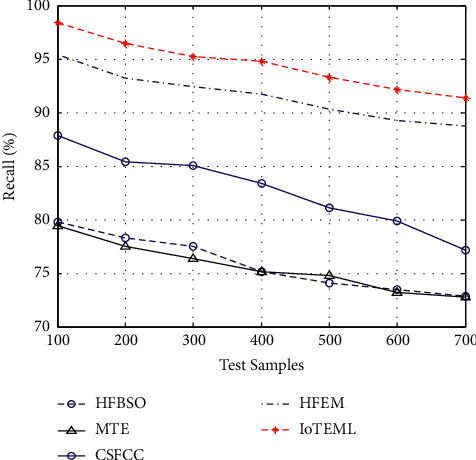
Measurement of tumour recall.

Figure 6 demonstrates the various measurement comparison of the tumour recall values between the existing HFBSO, MTE, CSFCC, HFEM, and proposed IOTEML.

### 4.4. Computation of Tumour F1-Score

It is measured by the average sample values of tumour precision and tumour recall of the samples.

(8) $$\begin{array}{c} \text{Tumour F}1-\text{score measurement}=\frac{2*\left(\text{recall}*\text{precision}\right)}{\left(\text{recall}+\text{precision}\right)}. \end{array}$$

**Figure 7:**
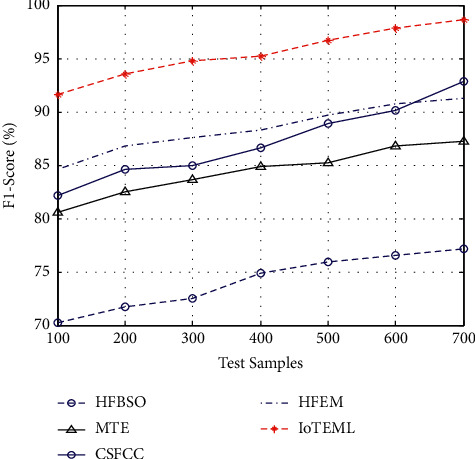
Measurement of tumour *F*1-score.

Figure 7 demonstrates the various measurement comparison of the tumour F1-score values between the existing HFBSO, MTE, CSFCC, HFEM, and proposed IOTEML.

### 4.5. Computation of Execution Time (ms)

The computation duration is nothing, but the time taken to calculate the prediction of two different images.

(9) $$\begin{array}{c} \text{Execution time}\left(ms\right)=\frac{\text{No}.\text{ of input samples}}{\text{computaion speed}}. \end{array}$$

**Figure 8:**
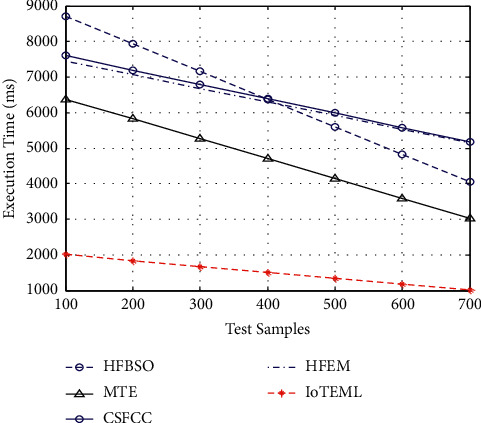
Measurement of execution time (ms).

Figure 8 demonstrates the various measurement comparison of the tumour accuracy values between the existing HFBSO, MTE, CSFCC, HFEM, and proposed IoTEML.

## 5. Conclusion

In this paper, various methods of diagnosing tumours are discussed, and its classification varies greatly depending on its type detection and accuracy. If diagnosed more accurately, the delay in calculating the size of the tumour and sending the tumour to the doctor will further increase the patient's risk. The state-of-the-art model with IoT further enhances time management by analyzing the size and type of tumours and making it possible to send the results immediately to the doctor without compromising accuracy. The proposed IoTEML was getting good accuracy, better precision, great recall rate, fine F1-score, and low computation duration compared with the existing HFBSO, MTE, CSFCC, and HFEM. Hence, the proposed IoT-based enhanced machine learning method was very accurate to identify the tumours with low time consumption. At a saturation point, the proposed IoT-EML model achieved 94.56% of accuracy, 94.12% of precision, 94.98% of recall, 95.12% of *F*1-score, and 1856 ms of execution time. These computations are very important in the medical field to diagnose the types of tumours in patients. It is especially helpful for physicians to obtain information about the nature of patients and their health from the place where IoT procedures were performed. The future enhancements of the proposed system include IoT-based consultation with the doctor and guided clinical examination, finding out the details of cancer and helping to get better treatment.

## Figures and Tables

**Algorithm 1:**
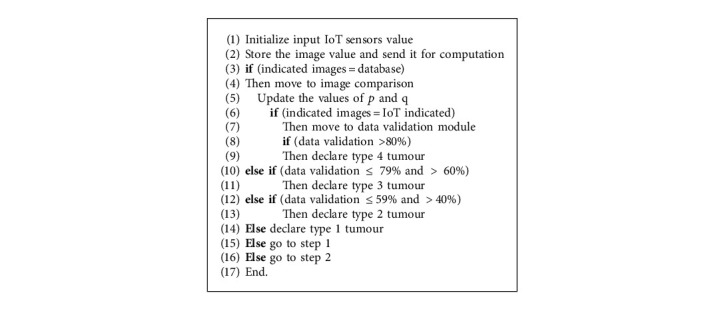
IoT-based enhanced machine learning.

**Table 1:** IoT sensor indication datasets.

Indications	IoT wearable indication
Head pain (HP)	(i) Blood pressure—high
	(ii) Body temperature—high
	(iii) Vomiting sensation

Vomiting sensation (VS)	(i) Blood pressure—high
	(ii) Body temperature—high
	(iii) Heart rate—high

Eye visual impact (EVI)	(i) Blood pressure—high
	(ii) Body temperature—high
	(iii) Heart rate—low
	(iv) Headache—medium

Abduction (AN)	(i) Blood pressure—high
	(ii) Heart rate—high

Walking issues (WI)	(i) Low number of walking foot works
	(ii) Lack of arm and leg coordination

Sleeping problems (SP)	(i) Less number of sleeping duration
	(ii) No deep sleeping

**Table 2:** Chances of tumour probabilities.

Chances of tumour probability	Level of result
Above 81%	Tumour confirmation: type 4
60% ≤ probability chance ≥ 80%	Tumour confirmation: type 3
40% ≤ probability chance ≥ 79%	Tumour confirmation: type 2
Below 40%	Tumour confirmation: type 1

## Data Availability

The data used to support the findings of this study are included within the article. Further data or information are available from the corresponding author upon request.
